# Bone morphogenetic protein-9 is a potent growth inhibitor of hepatocellular carcinoma and reduces the liver cancer stem cells population

**DOI:** 10.18632/oncotarget.12062

**Published:** 2016-09-16

**Authors:** Jae Woo Jung, So-Mi Yoon, Subin Kim, Yun-Hui Jeon, Byung-Hak Yoon, Su-Geun Yang, Min Kyoung Kim, Senyon Choe, Mario Meng-Chiang Kuo

**Affiliations:** ^1^ Protein Engineering Laboratory, Joint Center for Biosciences, Songdo Smart Valley, Yeonsu-gu, Incheon 406-840, South Korea; ^2^ Drug Discovery Collaboratory, University of California, San Diego, La Jolla, CA 92037, United States of America; ^3^ Department of New Drug Development, School of Medicine, Inha University, Incheon 400-712, South Korea; ^4^ Current address: Interdisciplinary Program in Genetic Engineering, Seoul National University, Seoul 151-742, South Korea; ^5^ Current address: Polaris Pharmaceuticals, Inc., San Diego, CA 92121, United States of America

**Keywords:** bone morphogenetic protein 9, hepatocellular carcinoma, TGF-beta, liver cancer stem cell, p21

## Abstract

The biological role of BMP-9 signaling in liver cancer remains dubious. To explore the potential use of BMP-9 signaling for anti-cancer therapy, we used recombinant human BMP-9, which we referred to as MB109, to study the effect on growth of fifteen hepatocellular carcinoma (HCC) cell lines. MB109 effectively inhibits the proliferation of nine HCC cells *in vitro*. The anti-proliferative effect was found to be induced by turning on p21 signaling, which caused survivin suppression and G0/G1 cell cycle arrest. ID3 was identified to be the mediator of the MB109-induced p21 expression. Blocking the activity of p38 MAPK diminished ID3 and p21 expression, indicating that MB109 signals through a p38 MAPK/ID3/p21 pathway to arrest cell cycle progression. Moreover, prolonged MB109 treatment suppressed the expression of five prominent liver cancer stem cell (LCSC) markers, including CD44, CD90, AFP, GPC3 and ANPEP. Xenograft model confirmed the anti-tumor and LCSC-suppression capability of MB109 *in vivo*. Contrary to ongoing efforts of suppressing BMP-9 signaling to inhibit angiogenesis of cancer tissue, these results demonstrate an unexpected therapeutic potential of MB109 to stimulate BMP-9 signaling for anti-cancer therapies.

## INTRODUCTION

Hepatocellular carcinoma (HCC) is the fifth most commonly occurring cancer and is the third leading cause of cancer deaths in the world [1]. Even with extensive research on diagnosis and treatments of HCC, prognosis remains unsatisfactory due to the extremely heterogeneous nature in their molecular pathogenesis and high risk of recurrence [2]. Resection, liver transplantation and several percutaneous treatments are curative therapies recommended for patients with early and intermediate HCC. Sorafenib, a small molecule targeting tyrosine protein kinases and Raf kinases to exert anti-angiogenic effect, is considered first-line treatment for patients with advanced, unresectable HCC, which improves survival for a median of about 3 months [2–4].

Transforming growth factor beta (TGF-β) superfamily ligands are extracellular cytokines which consist of more than 33 members, including TGF-βs, bone morphogenetic proteins (BMPs), growth and differentiation factors (GDFs), activins/inhibins, NODAL, myostatin and anti-Müllerian hormone (AMH). These ligands are able to activate canonical Smad-dependent, as well as non-canonical Smad-independent pathways, to exert different cellular responses in various tumor cells [5]. BMP-9 was originally cloned from a mouse liver cDNA library [6] and subsequently found to be predominantly expressed in liver tissue [7]. It circulates in human blood at biologically active concentrations of 2-12 ng/mL and regulates the growth and migration of endothelial cells, thus playing an essential role in physiological and pathological angiogenesis and lymphangiogenesis [8–12]. Several studies indicate that BMP-9 binds only to the ALK1 type I receptor and the BMPRII and ActRIIb type II receptors on cell surface to trigger downstream cellular responses [8, 13–16]. Therefore, attenuating BMP-9/ALK1 signaling is a currently favorable therapeutic strategy to inhibit angiogenesis and tumor growth [17, 18]. In contrast, in certain types of prostate and breast cancer cells, overexpression of BMP-9 inhibits proliferation [19, 20]. Additionally, expression analysis on human HCC tissue samples has shown that the innate BMP-9 expression level is positively correlated to tumor stage; therefore, BMP-9 has been proposed to be a proliferative and survival factor of HCC that induces epithelial-mesenchymal transition [21, 22].

Cancer stem cells (CSCs) have been implicated to have a major role in tumor formation, growth, metastasis, and chemoresistance [23, 24]. These cells have complex pathogenesis with sophisticated cross-talking and redundant signaling, many of which are shared with yet distinguish themselves from normal stem cells because of their pathogenic potentials. This complex characteristics makes it difficult to target CSCs for eradication and thus traditional single molecule/signaling targeting drugs have limited effect [25]. In contrast, differentiation therapy, which by definition induces reversion of malignant tumor cells to a more benign from, has shown future promise [24–26]. For instance, exogenous BMP-4 has been shown to promote differentiation of CD133^+^ liver cancer stem cells (LCSCs), thus blocking their transition to HCC [27]. By the same token, exogenous BMP-4 has also been shown to promote terminal differentiation, apoptosis and chemosensitization of colorectal cancer stem cells [28]. However, in this regard, the effect of other TGF-β superfamily ligands on the differentiation of LCSC has not been fully explored.

In this study, we investigated the therapeutic potential of the intrinsic BMP-9 signaling pathways in HCC cells. A recombinantly produced BMP-9 cytokine, termed MB109 [13], was used directly to study its cytotoxicity in HCC cells and to delineate its anti-cancer signaling pathway. Fifteen HCC cell lines were tested for time-course growth and dosage analyses. An anti-proliferative signaling pathway induced by MB109 was delineated in Hep3B cells. Moreover, we observed that prolonged exposure of Hep3B cells to MB109 suppressed the expression of five prominent LCSC markers during the course of treatment. Finally, a Hep3B xenograft model of NOD/SCID mice was carried out to verify the *in vitro* observations by injecting MB109 intraperitoneally and intravenously.

## RESULTS

### High dosage MB109 treatment inhibits the growth of a subset of HCC cells

**Figure 1:**
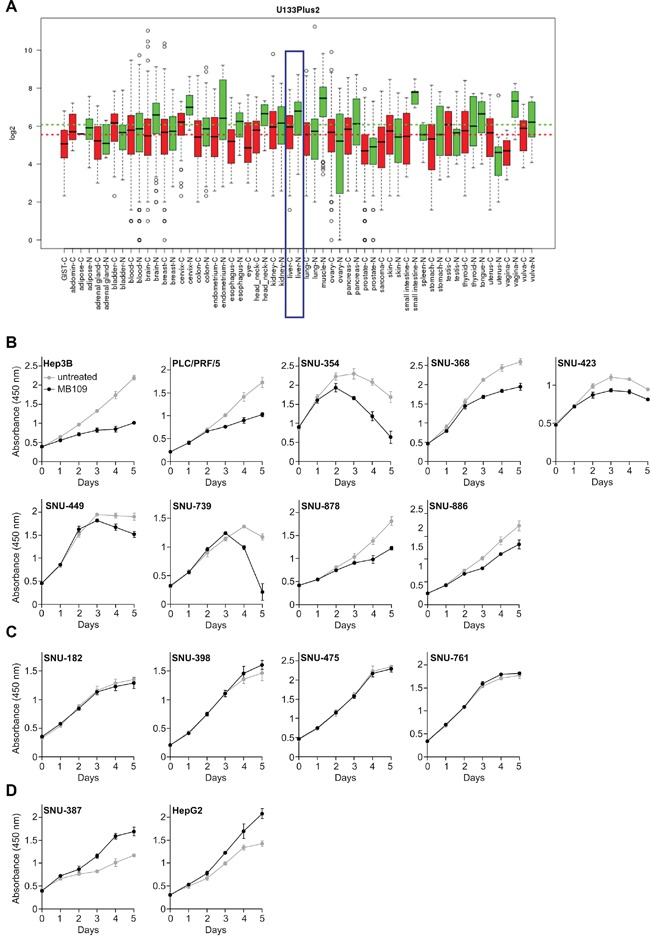
BMP-9 expression pattern analysis and MTT assay of 15 HCC cells in response to 200 ng/mL of MB109 treatment for 5 days **A.** Expression pattern of BMP-9 was analyzed in open data base “GENT”. The result was driven from 34000 samples of human cancer (red) and normal (green) tissues. The samples were profiled by Affymetrix U133plus2 platforms. Liver cancer and normal liver tissues are blue boxed. **B.** Nine HCC cell lines whose growth was inhibited by MB109 treatment. See also Supplementary Figure S1. **C.** Four HCC cell lines whose growth was not affected by MB109 treatment. See also Supplementary Figure S2. **D.** Two HCC cell lines whose growth was promoted by MB109 treatment. See also Supplementary Figure S3 All cells were grown in media containing 2% FBS, except SNU-368 (10%), SNU-423 (0.5%) and SNU-449 (10%). The representative data of at least three independent experiments are shown. All results are presented as mean±SD, n=4.

To explore the potential relationship between BMP-9 and HCC, we searched for BMP-9 expression in open data bases. From the Gene Expression across Normal and Tumor tissue database (GENT, http://mgrc.kribb.re.kr/GENT/, [29]), we found that BMP-9 is under-expressed in liver cancer tissues compared to normal liver tissues (Figure 1A, blue box). Moreover, we also noticed that the overall BMP-9 expression level in various cancer tissues (Figure 1A, red dash line) is lower than that in the normal tissues. (Figure 1A, green dash line).

Acknowledging the under-expressed state of BMP-9 in HCCs, we were encouraged to study the effects of external BMP-9 treatment on the growth of HCC cells. Fifteen HCC cell lines were tested for proliferation *in vitro* using recombinant mature form of human BMP-9, which we refer to as MB109 [13]. To identify the effective dosage that can affect the proliferation, broad range of concentration (0-2000 ng/mL) was screened for proliferation using MTT assay at various serum concentrations (Supplementary Figures S1-S3). For those cell lines whose growth was inhibited by MB109, the effective dosage was determined to be 200 ng/mL. Using determined effective dosage of MB109, MTT assay was performed on the fifteen HCC cells for 5 days (Figure 1B–1D). As shown in Figure 1B, 200 ng/mL of MB109 treatment significantly inhibited the growth of nine HCC cells including Hep3B, PLC/PRF/5, SNU-354, SNU-368, SNU-423, SNU-449, SNU-739, SNU-878 and SNU-886. Four other cells, SNU-182, SNU-398, SNU-475 and SNU-761, did not respond to MB109 treatment (Figure 1C), and the growth of the other two cells, SNU-387 and HepG2, were promoted by MB109 treatment (Figure 1D). These four non-responding and two growth promoted cell lines assure that 200 ng/mL of MB109 does not exert cytotoxicity. Moreover, the high effective dosage (200 ng/mL) of MB109 on growth inhibition did not correlate with the EC50 (~0.6 ng/mL) obtained from SMAD1/5/8 luciferase assay of Hep3B (Supplementary Figure S4). These results reveal that the high concentration treatment of MB109 causing growth inhibition of a certain subset of HCC cells is unlikely to be related to the canonical SMAD pathway.

### High dosage MB109 treatment induces p21 expression, survivin suppression and G0/G1 cell cycle arrest

**Figure 2:**
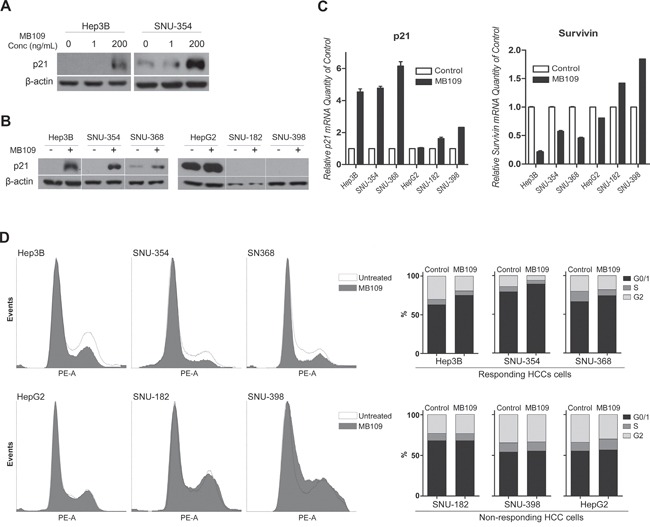
MB109 induces p21 expression, survivin suppression, and G0/G1 cell cycle arrest in HCC cells **A.** Western blot analyses of p21 in responding HCC cell lines, Hep3B and SNU-354, treated with 1 and 200 ng/ml MB109. **B.** Western blot analyses of p21 in responding (left panel) and non-responding (right panel) HCC cell lines after 24 hours of 200 ng/ml MB109 treatment. **C.** RT-PCR analysis of p21 and survivin expression at same condition explained in Figure 2B. **D.** Cell cycle determination by FACScan after PI staining. Left panel shows the raw data of FACScan. Right panel is the numerical conversion of the cell population in G1/0, S, and G2/M phases. The representative data of three independent experiments is shown. RT-PCR results are presented as mean±SD, n=3.

To identify molecular mechanism of the MB109-induced anti-proliferative effect, we focused on cell cycle regulating signals. When MB109-responding HCC cells, Hep3B and SNU-354, were exposed to 200 ng/mL of MB109 for 24 hours, p21 expression was dramatically induced, but 1 ng/mL did not have noticeable effect (Figure 2A). Same phenomenon was only observed in responding cell lines, Hep3B, SNU-354 and SNU-368 (Figure 2B, left panel), but not in non-responding cell lines (Figure 2B, right panel). RT-PCR analysis shows that MB109 treatment promoted p21 mRNA level only in responding cell lines, which reveals that it is a transcriptionally regulated event (Figure 2C left panel). In addition, MB109 suppressed the level of survivin mRNA only in responding cell lines (Figure 2C right panel). Since p21 and survivin are the key regulator of cell cycle progression, we then examined the cell cycle status of responding and non-responding cell lines over 48 hours of MB109 treatment at 200 ng/mL. The MB109 treatment significantly increased G0/G1 and decreased S and G2/M populations in responding cell lines, whereas noticeable change was not found in non-responding cell lines (Figure 2D). These results provide a possible explanation for a direct correlation among MB109-induced growth inhibition, p21 induction, survivin suppression and G0/G1 cell cycle arrest, implicating that the downstream effectors of the MB109 signaling pathway include p21 and survivin to exert G0/G1 cell cycle arrest.

### ID3 is a key mediator in MB109-induced p21 expression

**Figure 3:**
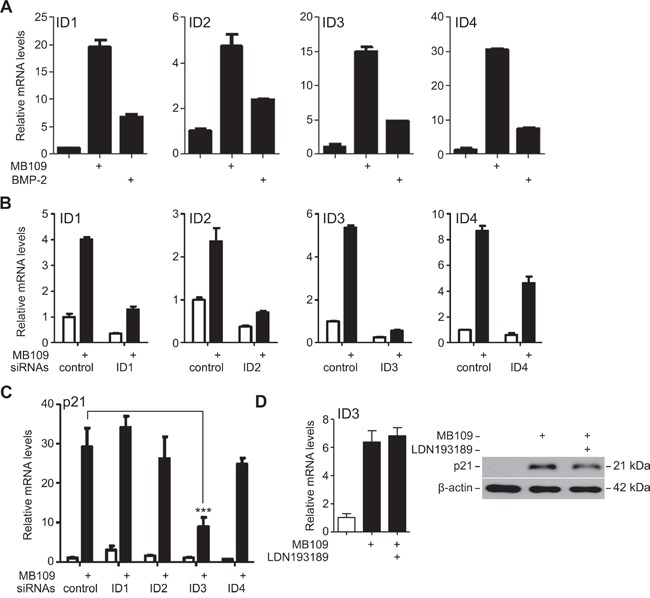
ID3 is involved in MB109-induced p21 expression in HCC cells **A.** RT-PCR analysis of ID1, ID2, ID3 and ID4 expression in Hep3B cells upon 200 ng/mL of MB109 and BMP-2 (control) treatments. **B.** Efficiency of siRNA knock-down of the IDs analyzed by RT-PCR. **C.** Expression of p21 mRNA was analyzed in each ID-KD background. Knock-down of ID3 was found to significantly attenuate MB109-induced p21 expression. (***: p<0.0001, one-way ANOVA with Dunnett's post-test) **D.** Analysis of MB109-induced ID3 mRNA (left panel) and p21 protein (right panel) expressions in the presence or absence of 50 nM LDN193189, a chemical inhibitor for ALK2/3/6. For all the experiments, MB109 was treated at 200 ng/ml for 24 hours. The representative data of three independent experiments is shown. RT-PCR results are presented as mean±SD, n=3.

To further investigate how MB109 may induce p21 expression, we focused on the inhibitor of DNA binding (ID) proteins (ID1, ID2, ID3 and ID4). IDs are dominant negative basic Helix-Loop-Helix (bHLH) family proteins, which bind on bHLH transcription factors to inhibit their binding to DNA and thus play a role in cell cycle regulation and oncogenesis [30, 31]. MB109 treatment induced strong mRNA expression of all four IDs in Hep3B cells (Figure 3A). To identify which IDs are involved in MB109-induced p21 expression, we knocked-down (KD) each ID individually using siRNA (Figure 3B). While the expression of all IDs was effectively knocked down, only the ID3-KD cells had significantly attenuated p21 expression upon MB109 treatment (Figure 3C).

The involvement of ALK2, 3 and 6 type I receptors in the MB109-induced p21 expression were tested by a chemical inhibitor, LDN193189, to block the signaling capability of ALK2/3/6 in order to study their involvement in MB109-induced ID3 and p21 expression. As shown in Figure 3D, blocking ALK2/3/6 had little effect on ID3 mRNA expression (left panel) and p21 protein expression (right panel) upon MB109 treatment, although LDN193189 was effective enough to suppress the SMAD1/5/8 signaling (Supplementary Figure S4). These results demonstrate that ID3 mediates the induction of p21 expression in the MB109 signaling pathway, which does not involve ALK2/3/6 type I receptors.

### p38 MAPK controls MB109-induced ID3 and p21 expression

**Figure 4:**
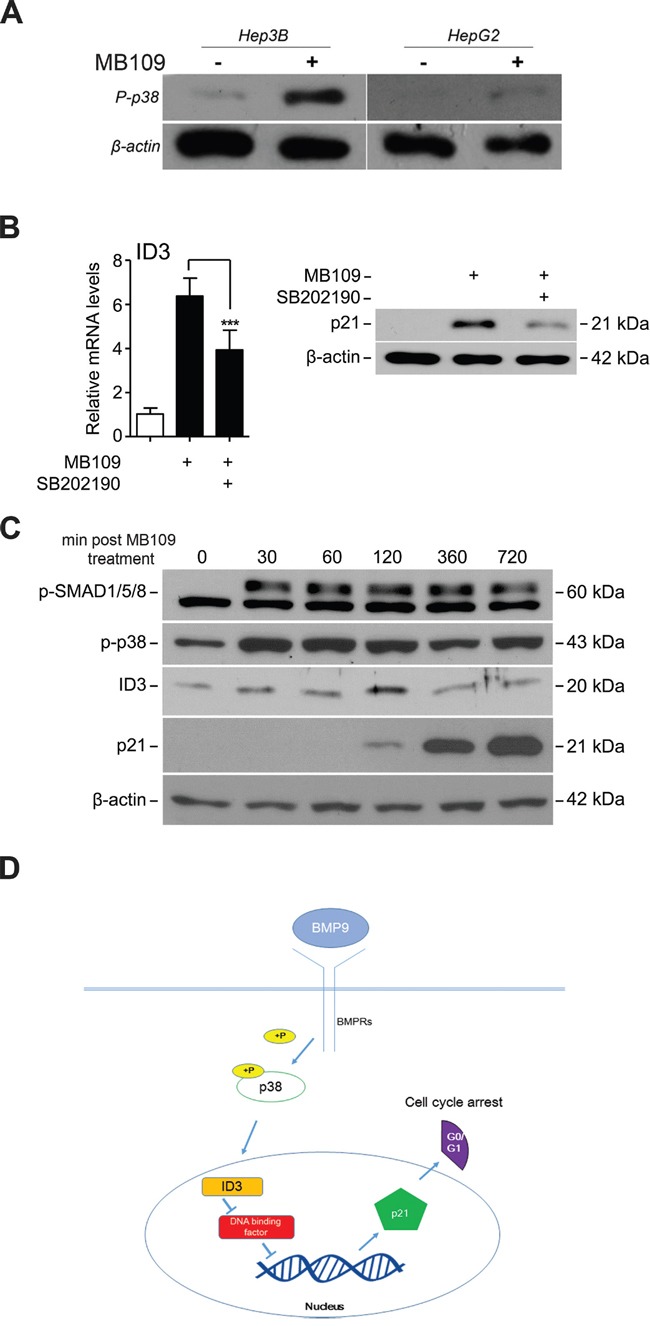
MB109 induces phosphorylation of p38 MAPK and controls ID3 and p21 expressions **A.** Hep3B and HepG2 cells were treated with 200 ng/ml of MB109 for 4 hours and analyzed by western blot. **B.** Analysis of MB109-induced ID3 mRNA (left panel) and p21 protein (right panel) expressions in the presence or absence of 50 nM SB202190, a chemical inhibitor for p38 MAPK activity. Cells were exposed to 200 ng/mL of MB109 for 4 hours. **C.** Western blot analysis of SMAD1/5/8 and p38 MAPK phosphorylations and ID3 and p21 expressions during 720 minutes of MB109 treatment. **D.** A working model of the anti-proliferative MB109 signaling pathway in HCCs. The representative results of three independent experiments are shown. RT-PCR results are presented as mean±SD, n=3.

To identify the upstream signaling molecules that induce ID3 expression, we focused on p38 mitogen-activated protein kinase (p38 MAPK), which is known to transduce SMAD-independent TGF-β signaling in various cell types [32]. As expected, high dosage MB109 treatment induced phosphorylation of p38 in Hep3B cells, but negligibly in HepG2 cells (Figure 4A). To find out whether p38 is an upstream regulator of MB109 induced ID3 and p21, a chemical inhibitor of p38, SB202190, was used to block p38 activity in Hep3B cells. Upon SB202190 treatment, MB109-induced ID3 mRNA (Figure 4B, left) and p21 protein expressions (Figure 4B, right) were both suppressed. These results demonstrate that the MB109-induced ID3/p21 expression in Hep3B cells requires p38 MAPK activity.

Most interestingly, the time courses expression analysis of the major signaling components over 720 minutes post-MB109 treatment shows that p38, as well as SMAD1/5/8, was phosphorylated efficiently within 30 minutes after MB109 treatment (Figure 4C). ID3 expression peaked at about 120 minutes, and then followed by dramatic induction of p21 expression. These data demonstrate that the MB109-induced G0/G1 cell cycle arrest is through p38/ID3/p21 signaling pathway, independent from SMAD signaling (Figure 4D).

### Prolonged MB109 treatment reduces cancer stem cell population

**Figure 5:**
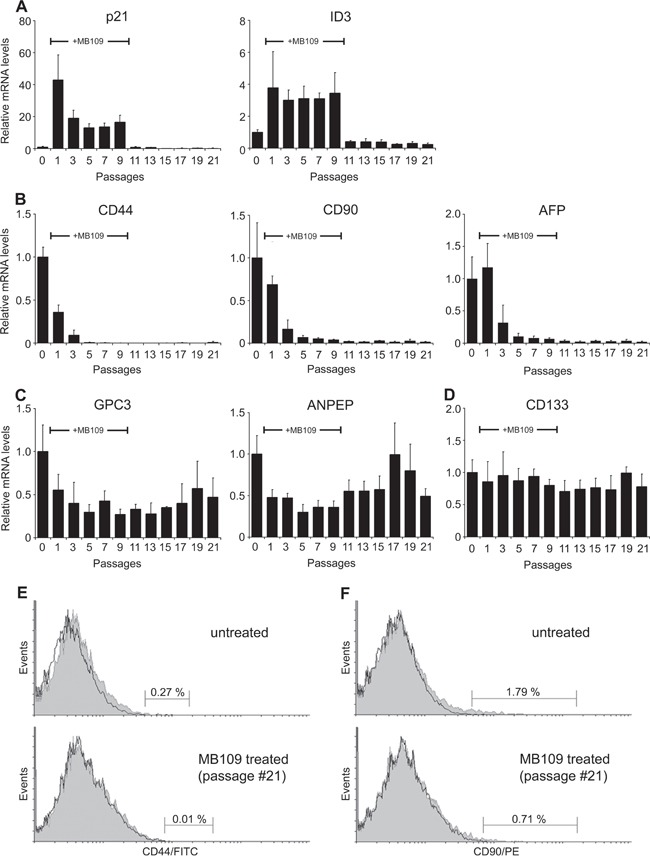
Prolonged MB109 treatment reduces different cancer stem cell populations in Hep3B cells **A.** RT-PCR analyses show that the expression levels of p21 (left panel) and ID3 (right panel) were drastically increased during prolonged MB109 treatment (passages #1–9), and were returned to basal levels right after MB109 was removed from the medium (passages #11-21). **B.** Expression levels of CD44 (left panel), CD90 (middle panel) and AFP (right panel) were reduced significantly and permanently by the prolonged MB109 treatment. **C.** Expression levels of GPC3 (left panel) and ANPEP (right panel) were moderately suppressed by MB109 treatment. **D.** Expression levels of CD133 were little or slightly suppressed by MB109 treatment. MB109 was treated at 200 ng/mL. RT-PCR results are presented as mean±SD, n=4. **E and F.** FACScan analyses show that the CD44^+^ (left panels) and CD90^+^ (right panels) populations were significantly reduced in the MB109-treated cells at passage #21 (bottom panels) as compared to the untreated Hep3B cells (top panels). Antibodies labeled and unlabeled cells are shown as gray areas and black lines, respectively. A representative data of four independent treatments is shown.

It has been reported that ID1 and ID3 govern self-renewal and cell cycle restriction on cancer stem cell (CSC) populations of colon cancer through regulation of p21 [33]. Since we discovered MB109's function to regulate ID3 and p21 expression to suppress the growth of HCC cells, we were encouraged to analyze the effect of MB109 on the CSC population. To investigate whether MB109 signaling plays a direct role on liver cancer stem cells (LCSCs), we selected six LCSC markers that are widely used by researchers and measured relative mRNA levels of these markers in responding HCC cell lines. Out of three cell lines, we determined Hep3B to be the valid candidate to observe the LCSC marker level change (Supplementary Figure S5). Hep3B cells were exposed to high dosage (200 ng/ml) of MB109 for 10 passages and then MB109 was removed from the medium for another 11 passages. The mRNA level of p21, ID3 and six prominent LCSC markers, including CD44, CD90, alpha-fetoprotein (AFP), glypican 3 (GPC3), alanine aminopeptidase (ANPEP or CD13) and CD133 [34–36] were monitored every other passage by RT-PCR in comparison to untreated group. Figure 5A, left panel, shows that p21 expression levels were peaked about 40-fold after MB109 treatment (passage #1) and stabilized at around 15-fold following the prolonged MB109 treatment (passages #3–9). Upon removal of MB109, p21 expression levels dropped back immediately to the basal levels (passage #11). ID3 expression levels also increased during the course of MB109 treatment, and returned to basal level right after the ligand removal (Figure 5A, right panel). For the LCSC markers, more than 10-fold reduction was observed on the expressions of CD44, CD90 and AFP during the MB109 exposure (Figure 5B). Upon removal of MB109, the expression of these three LCSC markers remained at reduced levels toward the end of the experiment. Expression levels of GPC3 and ANPEP (Figure 5C) were moderately reduced by prolonged MB109 treatment and gradually increased after MB109 was removed. In contrast, the prolonged MB109 treatment had little or slightly suppression effect on CD133 expression (Figure 5D).

To determine whether the reduced CD44 and CD90 mRNA levels resulted in the actual reduction of CD44^+^ and CD90^+^ cell populations, the MB109-treated cells at passage #21 were analyzed by flow cytometry. Indeed, MB109 treatment caused reduction of CD44^+^ population from 0.27% to 0.01% (Figure 5E) and reduction of CD90^+^ population from 1.79% to 0.71% (Figure 5F). All together, these results demonstrate that MB109 attenuates the expression of CD44, CD90 and AFP, which could be a result of selectively inhibiting CD44^+^, CD90^+^ and AFP^+^ populations.

### MB109 suppresses Hep3B cell growth and LCSC population in mouse xenograft model

**Figure 6:**
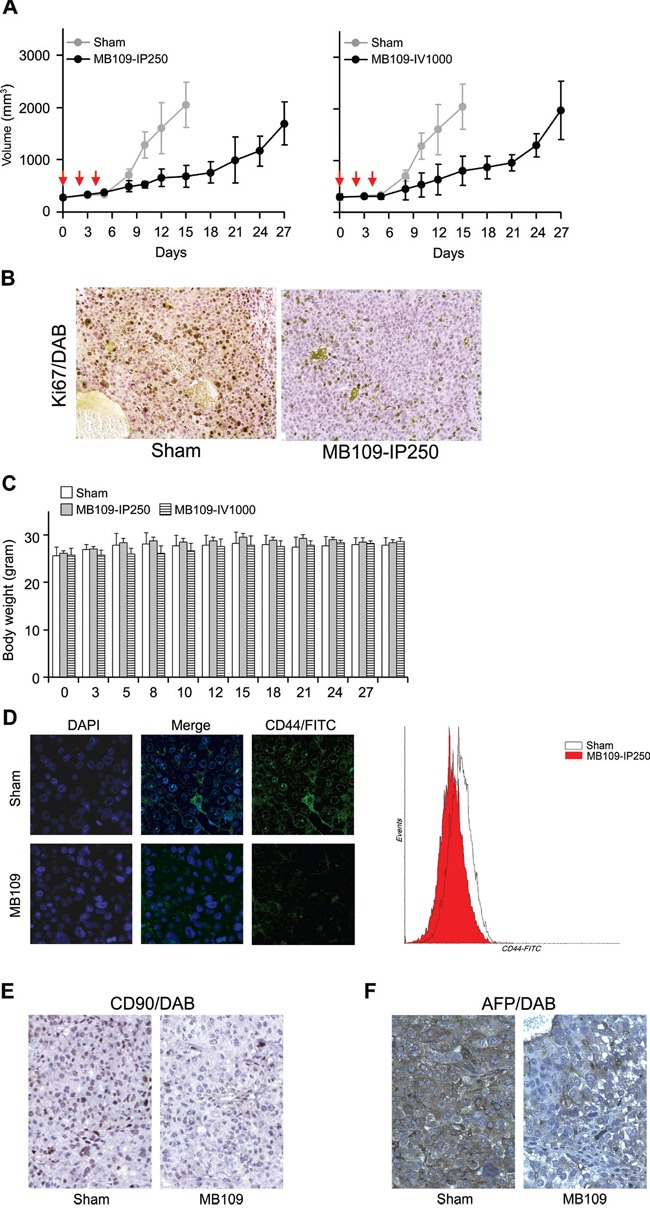
MB109 suppresses Hep3B cell growth and LCSC population in mouse xenograft model **A.** Time course analysis of tumor growth. MB109 was injected at 250 intraperitoneally (left panel), or 1000 μg/kg intravenously (right panel). Inhibition of tumor growth was observed in both experimental groups. Red arrows indicate the three time points of injection. The results are shown in means±SD (Sham, n=5; MB109-IP250, n=6; MB109-IV1000, n=4-5, one mouse was dead at day 8). **B.** IHC analysis of xenograft tumor from sham and MB109-IP250 group. Paraffin sectioned tissues were stained with proliferation marker Ki67/DAB (brown) and counterstained with hematoxylin (purple). **C.** No difference of the body weight was observed among the mice groups. **D.** Immuno-fluorescence images show that the expression of CD44 on the xenografted tumor tissue of the MB109-IP250 group was reduced as compared to that of the Sham group. CD44 antibodies were conjugated with FITC. Tumor tissues were nuclear counter stained with DAPI (left panel). CD44^+^ populations from the tumors were compared using FACScan (right panel). Immunohistochemistry images show that the expression of CD90 **E.** and AFP **F.** on the xenografted tumor tissue of the MB109-IP250 group was reduced as compared to that of the Sham group. Antibody bound regions of CD90 and AFP were visualized with DAB and counter stained with hematoxylin.

Since high dosage treatment of MB109 induced an anti-proliferative effect and LCSC suppression on HCC cells *in vitro*, we designed xenograft experiments to examine these effects *in vivo*. Hep3B cells were xenografted into non-obese diabetic/severe combined immunodeficiency (NOD/SCID) mice to form tumors of about 300 mm^3^, and then the mice were randomly assigned to 3 groups: Sham, MB109-IP250, and MB109-IV1000. Three single-dose injections were made either intraperitoneally (IP) (250 μg/kg for the MB109-IP250 group) or intravenously (IV) (1000 μg/kg for the MB109-IV1000 group) at day 0, 2 and 4. Both IP and IV injection method was utilized to observe the different effects due to slow and immediate delivery. In Figure 6A, the tumor growth of the two MB109 injected groups (black) was significantly hampered as compared to that of the Sham group (gray) in both MB109-IP250 and IV1000 groups. Immunohistochemistry (IHC) of proliferation marker Ki67 revealed reduced cancer cell proliferation in the tumor of MB109-IP250 group (Figure 6B). No significant body weight loss or cytotoxic death was observed due to high dosage injection of MB109, even for the 1000 μg/kg of MB109 IV-injected group (Figure 6C).

The xenografted tumors of the Sham and MB109-IP250 groups were subjected to immuno-fluorescence analysis and FACscan to visualize the expression of CD44 and CD44^+^ population. Significant reduction of CD44 expression and CD44^+^ population was observed in the tumor tissues of the MB109-IP250 group as compared to the Sham group (Figure 6D). Reduction of CD90 and AFP in the tumor tissue of MB109 group was also observed by immunohistochemistry analysis (Figure 6E and 6F, respectively). These results support the conclusion that the remission of tumor growth resulted from the reduction of LCSC markers by MB109-triggered signaling pathways.

## DISCUSSION

In this study, we did a comprehensive growth analysis on fifteen HCC cell lines by growing the cells in wide concentration ranges of serum and MB109. We found that the growth effect of MB109 varies with serum concentrations and HCC cell types. For instance, the growth of Hep3B, PLC/PRF/5 and SNU-878 cells were effectively inhibited by MB109 in all tested serum concentrations (0.1-10%, Supplementary Figure S1A, S1B, and S1H., respectively), whereas SNU-354 and SNU-449 cells were only inhibited when the cells were grown in certain ranges of serum concentrations (Supplementary Figure S1C and S1F., respectively). In addition, a dual growth effect was observed within certain HCC cells such as SNU-423 and SNU-739. Growth of SNU-423 cells was inhibited by MB109 when the cells were cultured at low serum concentrations (0.1-2%) and was promoted at high serum concentration (10%, Supplementary Figure S1E). In contrast, the growth of SNU-739 cells was promoted by MB109 at low serum concentrations (0.1-0.5%) and was inhibited at a moderate serum concentration (2%, Supplementary Figure S1G). Moreover, SNU-387 and HepG2 cells were only promoted by MB109 at low serum concentrations (0.1-2%, Supplementary Figure S3). Despite the diverse growth effects of MB109 on different HCC cells, these growth analyses demonstrated that high concentration of MB109 (200 ng/mL) consistently inhibited the growth of nine HCC cells, and thus highlights the notion that MB109 is able to trigger an anti-proliferative signaling pathway in a certain subset of HCC cells.

It remains unclear what defines a subset of HCC cells to be responsive to MB109. The fifteen HCC cell lines used in this study vary significantly in their genetic and etiological backgrounds, yet they have an apparent characteristic distinction related to hepatocarcinogenesis. Except HepG2, all fourteen other cells have integrated hepatitis B genes [37–41], for which we initially investigated whether the presence of the transcripts of Hepatitis B virus X (HBx) protein is a defining factor. We also investigated possible correlations between HBx expression and MB109 growth inhibition in the fourteen HCC cells, however, a clear correlation was not apparent. This led us to hypothesize that the MB109-induced growth inhibition may be independent of HBx expression. Our results support the notion that HCC often results from different genetic and epigenetic changes during years of development. To discover the genetic and epigenetic changes, we analyzed differential gene expression patterns of responding and non-responding groups from the open data bases (Supplementary Figure S6). The result from cancer cell line encyclopedia (CCLE, https://portals.broadinstitute.org/ccle [42]), shows that there are distinctively different gene expression patterns and copy number variations between the responding and non-responding groups of cell lines. Therefore, it is likely that the MB109-triggered anti-proliferative signaling pathway induced in the nine HCC cells is an evolutionary consequence of the long pathogenic process.

BMP-9 has been shown to activate Smad-dependent pathways through Smad1/5/8 to cause ID1 up-regulation and growth promotion in HepG2 liver cancer cells (Herrera et al., 2013). BMP-9 has also been shown to activate the ALK2/Smad1/Smad4 and the ALK4/Smad1 pathways to promote ovarian cancer cell proliferation [43] and to cause apoptosis in prostate cancer cells [20]. To characterize the anti-proliferative signaling pathways in HCC, we investigated a possible signaling mechanism in Hep3B cells by linking three known mechanistic axes of TGF-β signaling, p38/p21, TGF-β/p38 and TGF-β/p21. The importance of p38 in regulating p21 is well known, however, the underlying mechanism is unclear [44–46]. While it is clear that TGF-β ligands are able to activate p38 [47], it has long been questioned how TGF-β ligands, specially BMPs, activate p21. We found that in addition to the Smad-dependent pathways described above, MB109 can signal through a Smad-independent pathway, which involves p38 MAPK and ID3, to induce p21 expression and result in G0/G1 cell cycle arrest (Figure 4C). Although p21 induction correlates well with growth inhibition upon MB109 treatment in most other MB109-sensitive HCC cells (data not shown), it remains unclear whether the anti-proliferative MB109 signaling pathway that we delineated in this study applied generally to other MB109-sensitive HCC cells. Further investigation by RNA-sequencing may be instrumental to elucidate the complete landscape of the MB109-induced signaling network in HCC cells.

The most surprising and salient observation of our study is that high dosage MB109 treatment not only inhibits HCC cell proliferation, but also directly affects certain LCSC populations of Hep3B cells in prolonged ligand treatment. In order to reflect the population status of LCSC cells in the entire Hep3B pool, we devised an *in vitro* long-term treatment protocol. Noticeably, CD44, CD90 and AFP mRNA levels were prominently reduced during the initial 10 passages of MB109 treatment and continued to remain low even after the ligand was removed. Although CD44^+^ and CD90^+^ populations only constitute a small portion of Hep3B population (0.27 and 1.79%, respectively), a clear reduction of these LCSC cells was also observed by FACScan analysis. This result led us to consider that the Hep3B cells, or at least the CD44^+^, CD90^+^ and AFP^+^ LCSC populations, might have undergone cell differentiation upon prolonged exposure to MB109 as a possible explanation. Zhang et. al. have shown that exogenous BMP-4 treatment on PLC/PRF/5 HCC cells for 6 days was able to suppress CD133 expression and induce the differentiation of the sorted CD133^+^ cells [27]. It has also been shown recently that exposure of Hep3B cells to TGF-β1 for four weeks significantly reduced CD133^+^ and increased CD44^+^ populations [48]. In this study, we found that CD133 expression in Hep3B cells was little affected during the prolonged MB109 treatment, but CD44 expression was drastically reduced. To completely understand the role of BMP-9 signaling in LCSCs, elucidating the mechanistic differences of BMP-9 signaling between LCSCs and normal stem cells may provide valuable clue to develop an effective strategy for targeting LCSCs using MB109 as a therapeutic biologics.

## MATERIALS AND METHODS

### Recombinant proteins, chemicals, and antibodies

MB109 is a recombinantly expressed human BMP-9 cytokine. It contains a methionine residue in front of the mature domain of human BMP-9 (Ser320-Arg429, 110 residues). MB109 was prepared as described previously [13]. Recombinant BMP-2 was purchased from joint Protein Central (jointproteincentral.com, Incheon, Korea). The antibodies against p21, P-SMAD1/5/8 and P-p38 were purchased from Cell Signaling Technology (Massachusetts, USA), ID3 from Santa Cruz Biotechnology (California, USA), β-actin from Sigma Aldrich (Missouri, USA), CD90 from Novus Biologicals (CO, USA), AFP from Abcam (Cambridge, UK), PE-conjugated CD90 from BD Biosciences (New Jersey, USA) and FITC-conjugated CD44 from Miltenyi Biotec (Bergisch Gladbach, Germany). Chemical inhibitors, LDN193189 was purchased from Sellekchem (Texas, USA) and SB202190 was purchased from Sigma Aldrich (Missouri, USA). The cell culture media, fetal bovine serum (FBS), Penicillin-Streptomycin Solution (P/S) and Trypsin-EDTA were purchased from Hyclone (Utah, USA).

### Cell culture

Hep3B, PLC/PRF/5 and HepG2 cell lines were purchased from American Type Culture Collection (Virginia, USA) and SNU cell lines from Korean Cell Line Bank (Seoul, Korea). Cells were routinely cultured in DMEM (Hep3B, PLC/PRF/5 and HepG2) or RPMI-1640 (SNUs) medium supplemented with 10% FBS and 100 units/mL P/S at 37°C humidified atmosphere of 5% CO_2_. Cells were subcultured at 1:2 to 1:4 ratios every 3 or 4 days avoiding confluence.

### Cell proliferation assay

Cells at passages between #5 and #12 were plated in 96-well plates (BD, New Jersey, USA) at 750-3,000 cells/100 μL/well in stated conditions. After 16-20 hours, MB109 was treated at stated concentration. For time-course growth analysis, cell numbers were measured every 24 hours during 5 days of MB109 treatment. For dosage analysis, cell numbers were determined after 4 or 5 days of MB109 treatment. To determine cell numbers, Cell Counting Kit-8 (Dojindo Laboratories, Japan) and absorbance was measured at 450 nm.

### Western blot

Hep3B cells were plated in 12-well plates at 1 × 10^5^ cells/well in DMEM medium supplemented with 5% FBS without antibiotics. After 18 hours, MB109 was treated at final concentration of 200 μg/mL. After the indicated periods of MB109 treatment, cells were lysed using a Cell Lysis Buffer (Cell Signaling Technology, USA) supplemented with 1 mM PMSF and Phosphatase Inhibitor Cocktail (Roche, Basel, Switzerland). Western blot was performed using standard western blot protocol.

### Cell cycle determination assay

Hep3B cells were plated in 6-well plates at 2 × 10^5^ cells/well in DMEM medium supplemented with 10% FBS. After 12-18 hours, cells were washed twice with DPBS and serum-starved for 24 hours in the medium supplemented with 0.2% FBS. For MB109 treatment, the cell culture medium was replaced by a fresh medium supplemented with 5% FBS and 200 ng/mL of MB109. Cells were exposed to the ligand for 48 hours, harvested using Accutase (PAA Labs, Canada) and resuspended in 0.1% BSA in DPBS solution. Harvested cells were washed twice with 0.1% BSA-DPBS solution and fixed in absolute ethanol at −20°C. Fixed cells were washed twice in PBS and resuspended in propidium iodide (PI) solution (40 μg/mL in 3.8 mM sodium citrate, PBS). RNase A (10 μg/mL) solution was added to avoid non-specific RNA-PI binding. PI bound cells were analyzed using LSR II FACS system with FACS Diva software and Flowing Software 2.

### Real-time PCR

Cells were plated in 12-well plates at 2 × 10^5^cells/well. After ~20 hours, MB109 was added to a final concentration of 200 ng/mL and 24 hours later RNA was extracted. For long term MB109 treatment, Hep3B cells were grown in 100 mm Petri dishes for 26 passages. Two hundred ng/mL of MB109 were added to passages #6–15 (#1–10 in Figure 5), but not to passages #16–26 (#11–21 in Figure 5). Subcultures were made at 1:2 ratio when the cell density in each passage reached 80-90%. Four biological replicates were performed.

RNA was extracted using TRIsure (Bioline, UK) and cDNA synthesis was performed using ReverTra Ace qPCR RT Master Mix with gDNA remover (Toyobo, Japan). Analysis of mRNA expression was determined with quantitative real-time polymerase chain reaction (qRT-PCR) using Thunderbird SYBR qPCR Mix (Toyobo) and primers. The primer sequences are listed in Table 1.

**Table 1:** List of RT-PCR and siRNA oligonucleotides

Target gene	Sequence
RT-PCR	
Human β-actin	FWD-5′- CATGTACGTTGCTATCCAGGC REV-5′- CTCCTTAATGTCACGCACGAT
Human p21	FWD-5′- TGTCCGTCAGAACCCATGC REV-5′- AAAGTCGAAGTTCCATCGCTC
Human Survivin	FWD-5′-GATGACGACCCCATAGAGGAAC REV-5′-GGGTTAATTCTTCAAACTGCTTCT
Human CD44	FWD-5′- CTGCCGCTTTGCAGGTGTA REV-5′- CATTGTGGGCAAGGTGCTATT
Human CD133	FWD-5′- AGTCGGAAACTGGCAGATAGC REV-5′- GGTAGTGTTGTACTGGGCCAAT
Human CD90	FWD-5′-GGATACCAGGAGTTATTGGAGAAG REV-5′-CTTGGCTCTCCTGGATGTATTT
Human ANPEP	FWD-5′-GACCAGTACAGCGAGGTTAATG REV-5′-GGGTGGATCGGGTTATTATTGG
Human GPC3	FWD-5′-CCACCACTGAAACTGAGAAGAA REV-5′-GTTGTTGAGAATGGGCACATAAC
Human AFP	FWD-5′-GGATACCAGGAGTTATTGGAGAAG REV-5′-CTTGGCTCTCCTGGATGTATTT
Human ID1	FWD-5′-TTACGTGCTCTGTGGGTCTC REV-5′-CCCCCTAAAGTCTCTGGTGA
Human ID2	FWD-5′-ATGAAAGCCTTCAGTCCCGT REV-5′-TTCCATCTTGCTCACCTTCTT
Human ID3	FWD-5′-TCATCTCCAACGACAAAAGG REV-5′-ACCAGGTTTAGTCTCCAGGAA
Human ID4	FWD-5′-CGCTAAGCTACTGTCCAATCTC REV-5′-ATGAGACCAGAAATCACAGTACAA
siRNA	
Human ID1	FWD-5′-UCGCAUCUUGUGUCGCUGA(dTdT) REV-5′-UCAGCGACACAAGAUGCGA(dTdT)
Human ID2	FWD-5′-CUUACUUGGACUGUGAUAU(dTdT) REV-5′-AUAUCACAGUCCAAGUAAG(dTdT)
Human ID3	FWD-5′-CUGUAACAAUGCGAUGUAU(dTdT) REV-5′-AUACAUCGCAUUGUUACAG(dTdT)
Human ID4	FWD-5′-GUGACAUUUCAUACCAUGU(dTdT) REV-5′-ACAUGGUAUGAAAUGUCAC(dTdT)

Abundance of mRNA in each sample was determined by the difference of the cycle threshold (Cq) value between each genes and β-actin, ΔCq. Relative mRNA level was defined as 2^−ΔΔCq^, where ΔΔCq=ΔCq_sample_-ΔCq_control_, which reflects changes of mRNA expression level between treated and untreated cells.

### mRNA knock-down using siRNA

Cells were reverse transfected with siRNA duplex, complexed with Lipofectamine RNAiMAX reagent (Invitrogen, California, USA) in serum free media as specified in manufacturer's instruction. Three independent siRNA sequences for each gene were ordered from Bioneer (Korea) and their sequences were listed in Table I. Negative controls were transfected with *AccuTarget* Negative control siRNA (Bioneer, Korea).

### FACScan analysis

For flow cytometry, Hep3B cells were harvested, washed in FACS buffer (1% BSA and 0.1% sodium azide in PBS) and resuspended in the same buffer at 1 × 10^6^ cells/mL in 100 μL. CD44-FITC and CD90-PE antibodies were diluted according to manufacturer's recommendations and used to label the cells by incubating in dark for 40 to 60 minutes on ice. Cells were washed three times with 0.5 mL of FACS buffer and resuspended to the same volume for analysis. BD biosciences LSR (USA, CA) was used to collect 10,000 – 20,000 total events. Analysis and population calculations were performed using Flowing Software 2.

### *In vivo* xenograft

Hep3B cells were subcutaneously injected into dorsolateral flank of 7-week-old NOD/SCID mice at 5 × 10^6^ cells/mouse in 200 μL of 50% cells (in PBS)+50% Matrigel. After ~3 weeks of cell injection, the mice with tumor volume between 200–300 mm^3^ were randomly assigned to four groups: Sham (n=5), MB109-IP(250) (n=6), MB109- IP(1000) (n=6) and MB109-IV(1000) (n=5). Three single-dose injections of MB109 were made intraperitoneally (250 or 1000 μg/kg body weight for the MB109-IP250 and MB109-IP1000 groups) and intravenously (1000 μg/kg for the MB109-IV1000 group) at day 0, 2, and 4. The Sham group was injected intraperitoneally with 5 mL/kg body weight of PBS. Tumor volume was measured every 2 or 3 days, and calculated based on an equation of (long axis)×(short axis)^2^/2. All animal experiments were done under the guideline of AAALAC (Association for Assessment and Accreditation of Laboratory Animal Care International). The procedures were performed at the Inha University hospital, NCEED (Incheon, South Korea) and protocols were approved by Institutional Animal Care and Use Committee (IACUC No. NHA130816-225-1) at Inha University hospital in South Korea. All the experiments were designed to minimize the animal suffering.

### Immunohistochemistry

Collected xenograft tumors were fixed in formaldehyde, embedded in paraffin and sectioned on coated slides. Sections were deparaffinized using xylene and hydrated in graded ethanol series (100%, 95%, 75% and 40%). Antigen was retrieved by microwave in 10 mM sodium citrate pH 6.0. Sections were blocked with 0.3% hydrogen peroxide and 1% BSA in TBS. CD90 and AFP primary antibodies were diluted in 1% BSA in TBS at 1:100 and 1:50 ratio, respectively, and incubated ~16 hours at 4°C. For HRP detection, ImmPRESS polymerized reporter enzyme staining system and DAB peroxidase substrate kit (Vector Laboratories, California, USA) was used for detection. Sections were counter stained using Harris Hematoxylin (BBC Biochemical, London, UK) for the appropriate period. For fluorescence detection, FITC-conjugated CD44 antibody was used and nuclear counter stained with DAPI (Sigma). Areas of similar cell morphology and density were compared.

## SUPPLEMENTARY MATERIALS FIGURES


