# Supplementary material for: Bone morphogenetic protein-9 is a potent growth inhibitor of hepatocellular carcinoma and reduces the liver cancer stem cells population
Source: Oncotarget. 2016 Sep 16;7(45):73754–68. doi: 10.18632/oncotarget.12062 (PMC5342011; doi:10.18632/oncotarget.12062)
Supplement: Supplementary file 1 [file oncotarget-07-73754-s001.pdf]

Bone morphogenetic protein-9 is a potent growth inhibitor of hepatocellular carcinoma and reduces the liver cancer stem cells population

SUPPLEMENTARY FIGURES

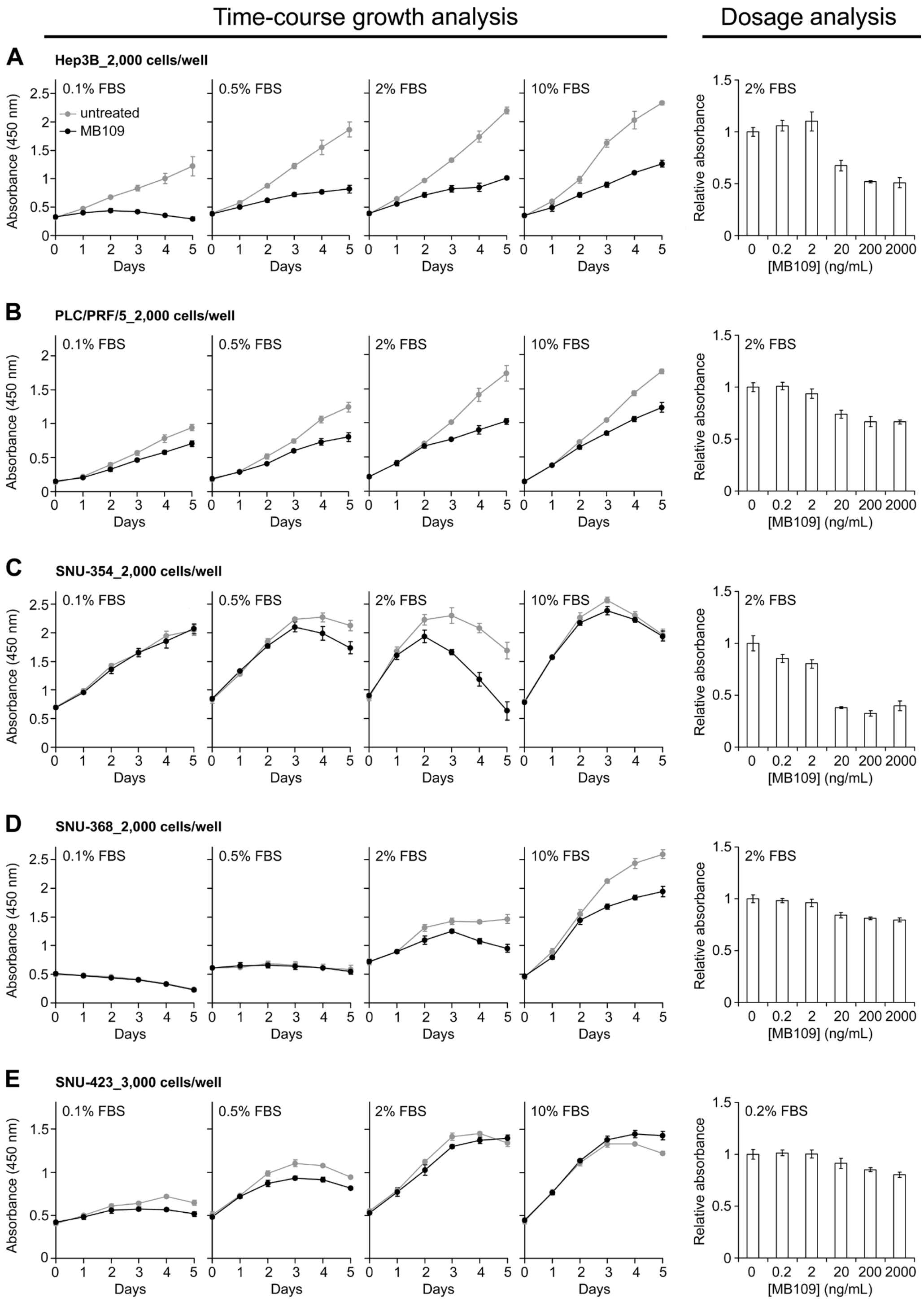

(Continued)

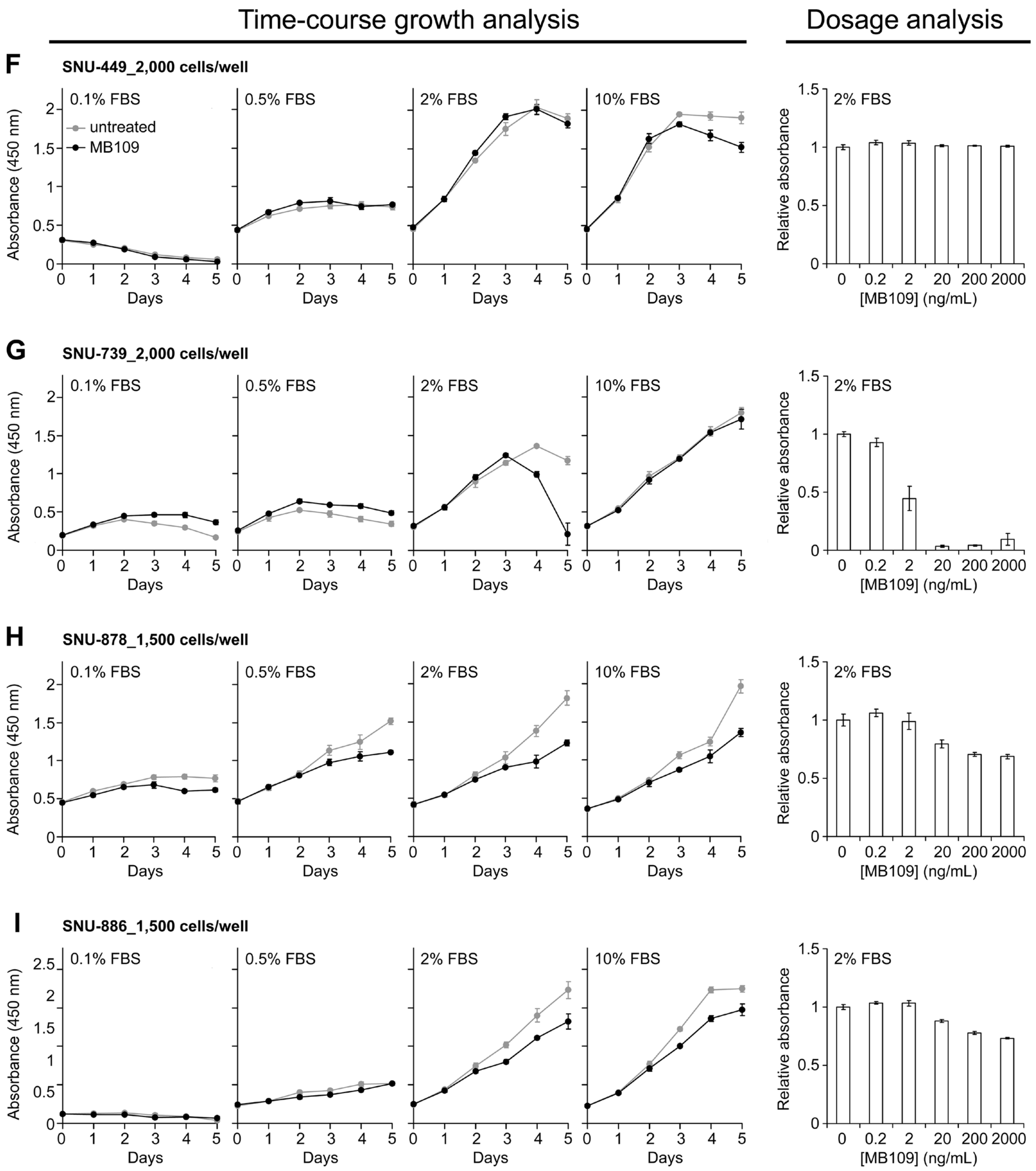

Supplementary Figure S1: Cell proliferation data of the nine HCC cells that can be inhibited by MB109 treatment. Panels of the left four columns are time-course growth analysis. Cells were cultured in media containing 0.1, 0.5, 2 and 10% FBS. Two hundred ng/ml of MB109 was treated for five days, and the cell numbers were measured every day. Panels of the right column are dosage analysis. Cell numbers were measured after four or five days of ligand treatment. All results are present as mean±SD, n=3-4.

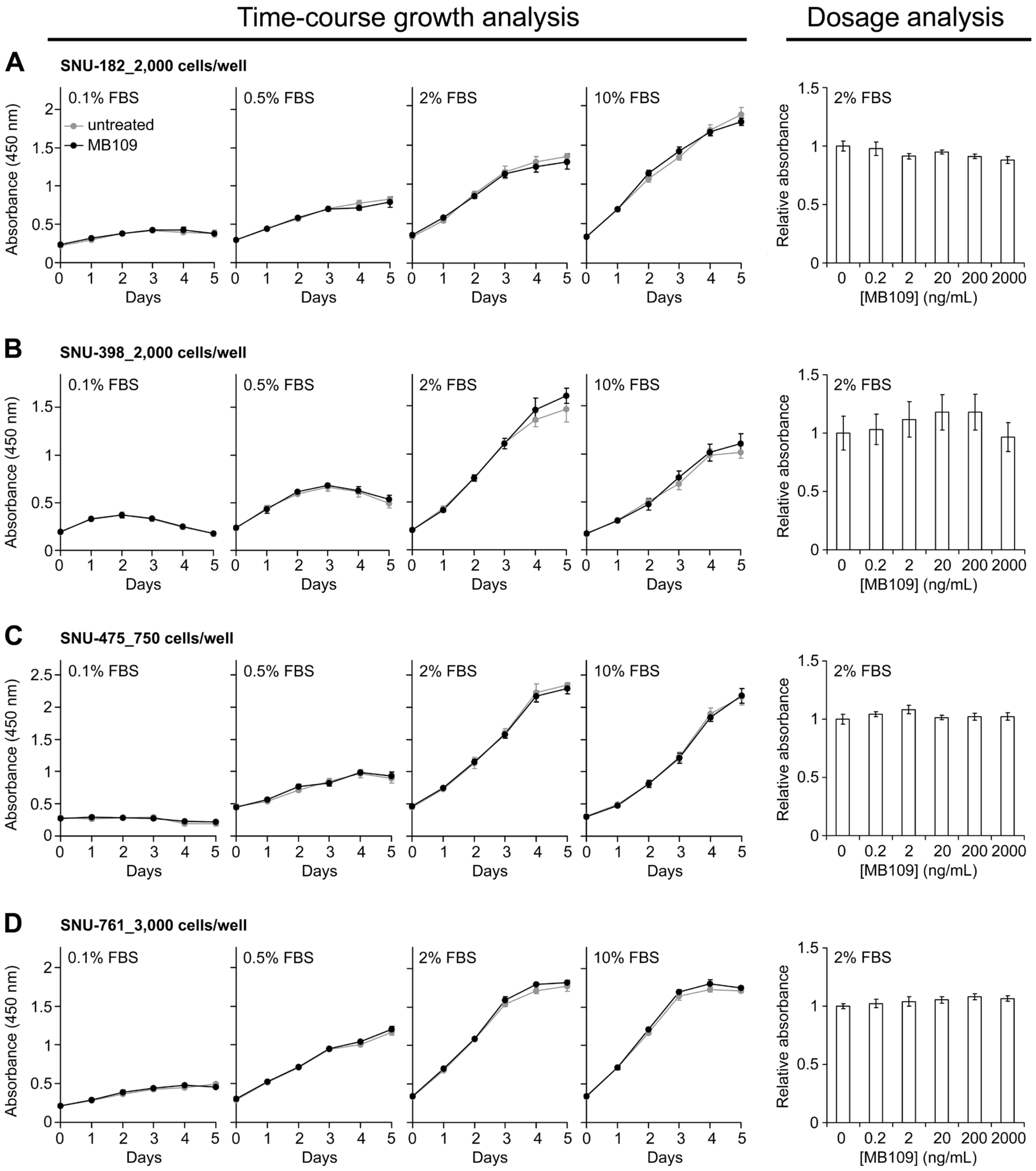

Supplementary Figure S2: Cell proliferation data of the four HCC cells that do not response to MB109 treatment in all tested FBS concentrations. All results are presented as mean±SD, n=3-4.

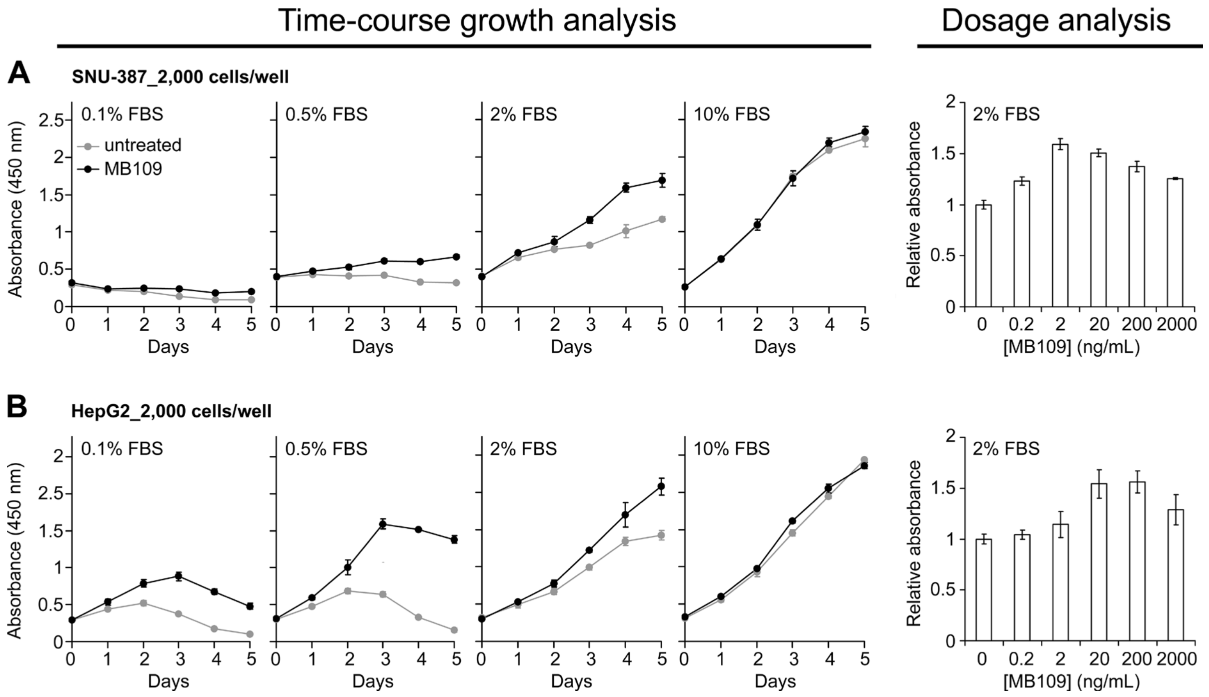

Supplementary Figure S3: Cell proliferation data of the two HCC cells that can be promoted by MB109 treatment. All results are presented as mean±SD, n=3-4.

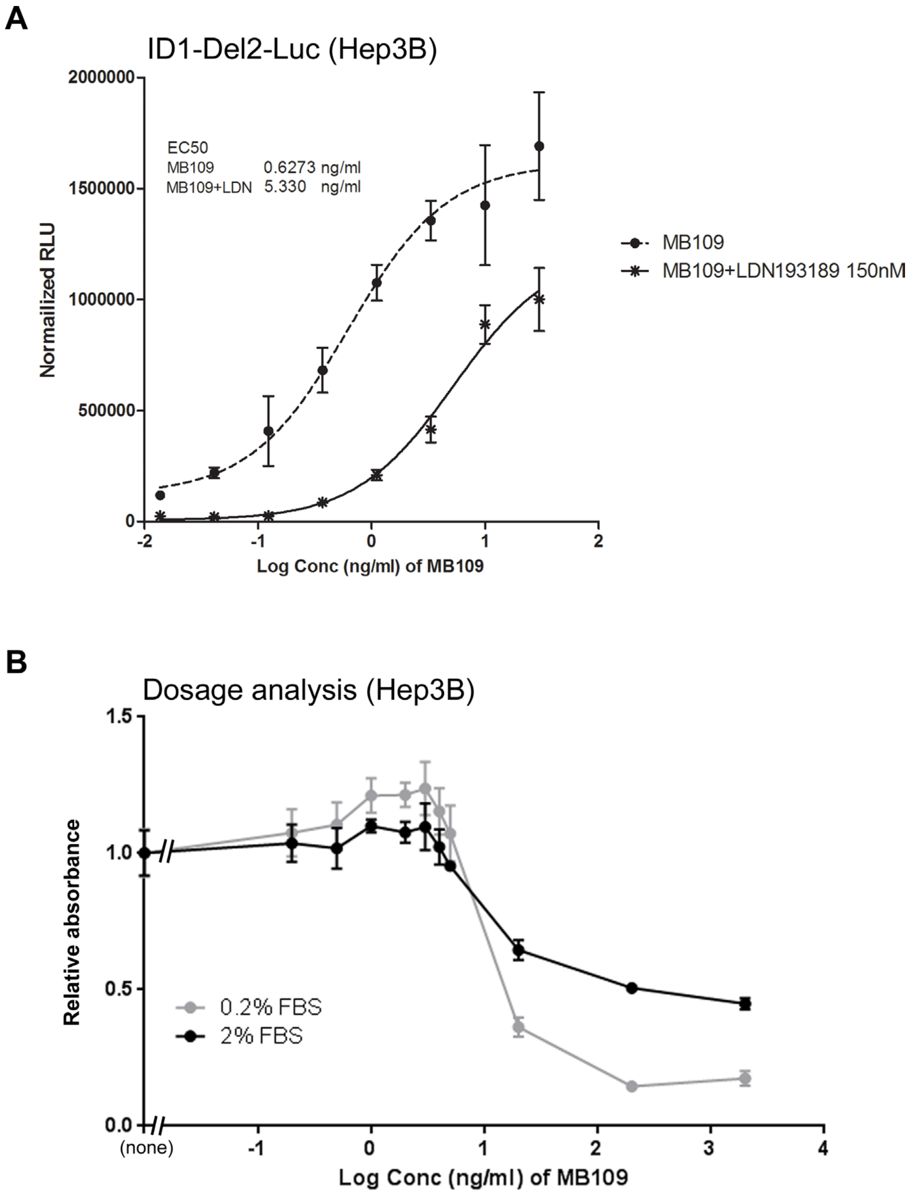

Supplementary Figure S4: ID1-Luciferase assay and dosage analysis of MB109 on Hep3B cells. A. Luciferase assay was performed by measuring SMAD1/5/8 signaling capacity of MB109. Serially diluted MB109 was treated with or without 150 nM of BMP receptor inhibitor, LDN193189. Obtained relative luciferase unit data were normalized with co-transfected beta-galactosidase enzyme activity. Data are shown in mean±SEM, n=5. **B.** For dosage analysis, the cells were cultured in media containing 0.2% and 2% FBS. MB109 was treated at 0, 0.2, 0.5, 1, 2, 3, 4, 5, 20, 200 and 2000 ng/mL for five days. Data are shown in mean±SD, n=4.

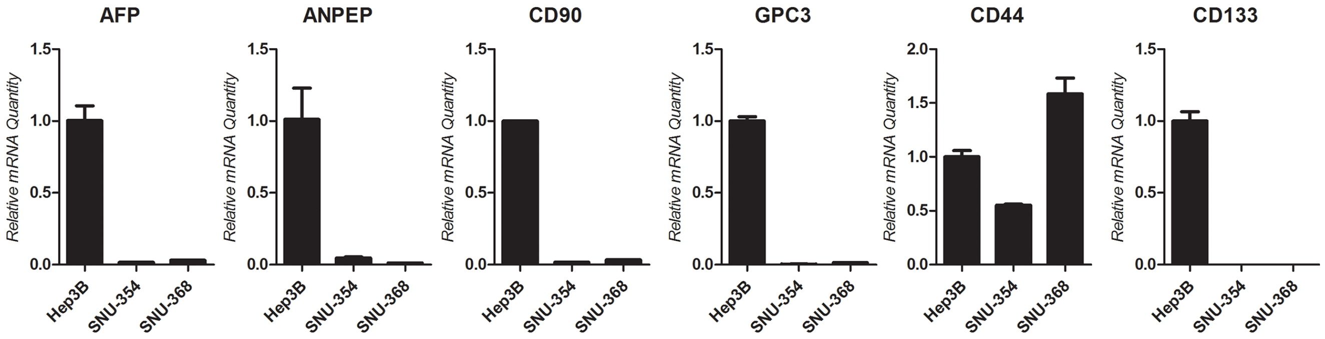

Supplementary Figure S5: Basal expression analysis of six prominent LCSC markers in three MB109 responding HCC cell lines. The mRNA expression levels were analyzed using RT-PCR. Shown data are representative of at least three independent experiments. Data are shown in mean±SD, n=5.

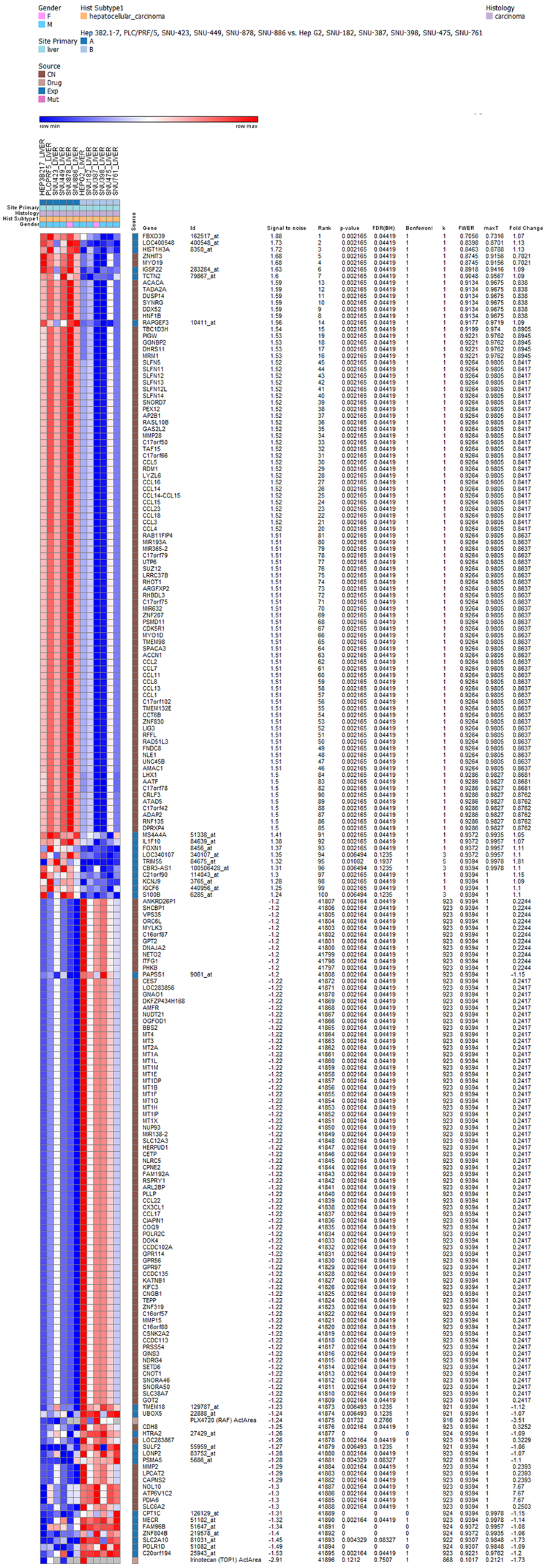

Supplementary Figure S6: Differential expression analysis of MB109 responsive vs non-responsive HCC cell lines. Differential gene expression analysis of six MB109 responsive vs non-responsive HCC cell lines were performed from cancer cell line encyclopedia (CCLE, https://portals.broadinstitute.org/ccle/home).
